# SpadaHC: a database to improve the classification of variants in hereditary cancer genes in the Spanish population

**DOI:** 10.1093/database/baae055

**Published:** 2024-07-04

**Authors:** José M Moreno-Cabrera, Lidia Feliubadaló, Marta Pineda, Patricia Prada-Dacasa, Mireia Ramos-Muntada, Jesús Del Valle, Joan Brunet, Bernat Gel, María Currás-Freixes, Bruna Calsina, Milton E Salazar-Hidalgo, Marta Rodríguez-Balada, Bàrbara Roig, Sara Fernández-Castillejo, Mercedes Durán Domínguez, Mónica Arranz Ledo, Mar Infante Sanz, Adela Castillejo, Estela Dámaso, José L Soto, Montserrat de Miguel, Beatriz Hidalgo Calero, José M Sánchez-Zapardiel, Teresa Ramon Y Cajal, Adriana Lasa, Alexandra Gisbert-Beamud, Anael López-Novo, Clara Ruiz-Ponte, Miriam Potrony, María I Álvarez-Mora, Ana Osorio, Isabel Lorda-Sánchez, Mercedes Robledo, Alberto Cascón, Anna Ruiz, Nino Spataro, Imma Hernan, Emma Borràs, Alejandro Moles-Fernández, Julie Earl, Juan Cadiñanos, Ana B Sánchez-Heras, Anna Bigas, Gabriel Capellá, Conxi Lázaro

**Affiliations:** Hereditary Cancer Program, Catalan Institute of Oncology, Institut d’Investigació Biomèdica de Bellvitge-IDIBELL-ONCOBELL, L’Hospitalet de Llobregat, Barcelona 08908, Spain; Centro de Investigación Biomédica en Red Cáncer (CIBERONC), Instituto de Salud Carlos III, Monforte de Lemos, 3-5, Madrid, 28029, Spain; Oncology Data Analytics Program (ODAP), Catalan Institute of Oncology (ICO), L’Hospitalet del Llobregat, Barcelona 08908, Spain; Hereditary Cancer Program, Catalan Institute of Oncology, Institut d’Investigació Biomèdica de Bellvitge-IDIBELL-ONCOBELL, L’Hospitalet de Llobregat, Barcelona 08908, Spain; Centro de Investigación Biomédica en Red Cáncer (CIBERONC), Instituto de Salud Carlos III, Monforte de Lemos, 3-5, Madrid, 28029, Spain; Hereditary Cancer Program, Catalan Institute of Oncology, Institut d’Investigació Biomèdica de Bellvitge-IDIBELL-ONCOBELL, L’Hospitalet de Llobregat, Barcelona 08908, Spain; Centro de Investigación Biomédica en Red Cáncer (CIBERONC), Instituto de Salud Carlos III, Monforte de Lemos, 3-5, Madrid, 28029, Spain; Hereditary Cancer Program, Catalan Institute of Oncology, Institut d’Investigació Biomèdica de Bellvitge-IDIBELL-ONCOBELL, L’Hospitalet de Llobregat, Barcelona 08908, Spain; Centro de Investigación Biomédica en Red Cáncer (CIBERONC), Instituto de Salud Carlos III, Monforte de Lemos, 3-5, Madrid, 28029, Spain; Hereditary Cancer Program, Catalan Institute of Oncology, Institut d’Investigació Biomèdica de Bellvitge-IDIBELL-ONCOBELL, L’Hospitalet de Llobregat, Barcelona 08908, Spain; Centro de Investigación Biomédica en Red Cáncer (CIBERONC), Instituto de Salud Carlos III, Monforte de Lemos, 3-5, Madrid, 28029, Spain; Hereditary Cancer Program, Catalan Institute of Oncology, Institut d’Investigació Biomèdica de Bellvitge-IDIBELL-ONCOBELL, L’Hospitalet de Llobregat, Barcelona 08908, Spain; Centro de Investigación Biomédica en Red Cáncer (CIBERONC), Instituto de Salud Carlos III, Monforte de Lemos, 3-5, Madrid, 28029, Spain; Hereditary Cancer Program, Catalan Institute of Oncology, Institut d’Investigació Biomèdica de Bellvitge-IDIBELL-ONCOBELL, L’Hospitalet de Llobregat, Barcelona 08908, Spain; Centro de Investigación Biomédica en Red Cáncer (CIBERONC), Instituto de Salud Carlos III, Monforte de Lemos, 3-5, Madrid, 28029, Spain; Hereditary Cancer Group, Program for Predictive and Personalized Medicine of Cancer, Germans Trias i Pujol Research Institute (PMPPC-IGTP), Campus Can Ruti, Ctra de Can Ruti, Camí de les Escoles, s/n, Badalona 08916, Spain; Familial Cancer Clinical Unit, Human Cancer Genetics Program, Spanish National Cancer Research Centre (CNIO), Melchor Fernández Almagro, 3, Madrid 28029, Spain; Familial Cancer Clinical Unit, Human Cancer Genetics Program, Spanish National Cancer Research Centre (CNIO), Melchor Fernández Almagro, 3, Madrid 28029, Spain; Familial Cancer Clinical Unit, Human Cancer Genetics Program, Spanish National Cancer Research Centre (CNIO), Melchor Fernández Almagro, 3, Madrid 28029, Spain; Institut d’Oncologia de la Catalunya Sud (IOCS), Hospital Universitari Sant Joan de Reus (HUSJR), Institut d’Investigació Sanitària Pere Virgili (IISPV), Universitat Rovira i Virgili (URV), Dr. Josep Laporte, 2, Reus 43204, Spain; Institut d’Oncologia de la Catalunya Sud (IOCS), Hospital Universitari Sant Joan de Reus (HUSJR), Institut d’Investigació Sanitària Pere Virgili (IISPV), Universitat Rovira i Virgili (URV), Dr. Josep Laporte, 2, Reus 43204, Spain; Institut d’Oncologia de la Catalunya Sud (IOCS), Hospital Universitari Sant Joan de Reus (HUSJR), Institut d’Investigació Sanitària Pere Virgili (IISPV), Universitat Rovira i Virgili (URV), Dr. Josep Laporte, 2, Reus 43204, Spain; Cancer Genetics Group, Unit of Excellence Institute of Biomedicine and Molecular Genetics, University of Valladolid-Spanish National Research Council (IBGM, UVa- CSIC), Sanz y Fores, 3, Valladolid 47003, Spain; Cancer Genetics Group, Unit of Excellence Institute of Biomedicine and Molecular Genetics, University of Valladolid-Spanish National Research Council (IBGM, UVa- CSIC), Sanz y Fores, 3, Valladolid 47003, Spain; Cancer Genetics Group, Unit of Excellence Institute of Biomedicine and Molecular Genetics, University of Valladolid-Spanish National Research Council (IBGM, UVa- CSIC), Sanz y Fores, 3, Valladolid 47003, Spain; Unidad de Genética Molecular, Hospital General Universitario de Elche. Fundación para el Fomento de la Investigación Sanitaria y Biomédica de la Comunitat Valenciana (FISABIO), Av. de Catalunya, 21, Elche 03203, Spain; Unidad de Genética Molecular, Hospital General Universitario de Elche. Fundación para el Fomento de la Investigación Sanitaria y Biomédica de la Comunitat Valenciana (FISABIO), Av. de Catalunya, 21, Elche 03203, Spain; Unidad de Genética Molecular, Hospital General Universitario de Elche. Fundación para el Fomento de la Investigación Sanitaria y Biomédica de la Comunitat Valenciana (FISABIO), Av. de Catalunya, 21, Elche 03203, Spain; Laboratorio de cáncer hereditario, Servicio de Bioquímica clínica-Análisis clínicos, Hospital Universitario 12 de Octubre, Av. de Córdoba, s/n, Madrid 28041, Spain; Laboratorio de cáncer hereditario, Servicio de Bioquímica clínica-Análisis clínicos, Hospital Universitario 12 de Octubre, Av. de Córdoba, s/n, Madrid 28041, Spain; Laboratorio de cáncer hereditario, Servicio de Bioquímica clínica-Análisis clínicos, Hospital Universitario 12 de Octubre, Av. de Córdoba, s/n, Madrid 28041, Spain; Familial Cancer Clinic, Medical Oncology, Hospital de la Santa Creu i Sant Pau, Sant Quintí, 89, Barcelona 08041, Spain; Genetics Department, Hospital de la Santa Creu i Sant Pau, Sant Quintí, 89, Barcelona 08041, Spain; Biomedical Network Research Centre On Rare Diseases (CIBERER), Instituto de Salud Carlos III, Monforte de Lemos, 3-5, Madrid 28029, Spain; Institut de Recerca Sant Pau (IR Sant Pau), Sant Quintí, 77, Barcelona 08041, Spain; Fundación Pública Galega de Medicina Xenómica (SERGAS), Instituto de Investigación Sanitaria de Santiago, Grupo de Medicina Xenómica-USC, Av. Barcelona, s/n, Santiago de Compostela 15706, Spain; Biomedical Network Research Centre On Rare Diseases (CIBERER), Instituto de Salud Carlos III, Monforte de Lemos, 3-5, Madrid 28029, Spain; Fundación Pública Galega de Medicina Xenómica (SERGAS), Instituto de Investigación Sanitaria de Santiago, Grupo de Medicina Xenómica-USC, Av. Barcelona, s/n, Santiago de Compostela 15706, Spain; Biomedical Network Research Centre On Rare Diseases (CIBERER), Instituto de Salud Carlos III, Monforte de Lemos, 3-5, Madrid 28029, Spain; Biochemistry and Molecular Genetics Department, Hospital Clinic of Barcelona and Fundació de Recerca Clínic Barcelona-Institut d’Investigacions Biomèdiques August Pi i Sunyer (FRCB-IDIBAPS), University of Barcelona, Rosselló, 149, Barcelona 08036, Spain; Biomedical Network Research Centre On Rare Diseases (CIBERER), Instituto de Salud Carlos III, Monforte de Lemos, 3-5, Madrid 28029, Spain; Biochemistry and Molecular Genetics Department, Hospital Clinic of Barcelona and Fundació de Recerca Clínic Barcelona-Institut d’Investigacions Biomèdiques August Pi i Sunyer (FRCB-IDIBAPS), University of Barcelona, Rosselló, 149, Barcelona 08036, Spain; Biomedical Network Research Centre On Rare Diseases (CIBERER), Instituto de Salud Carlos III, Monforte de Lemos, 3-5, Madrid 28029, Spain; Departamento de Genética y Genómica, Hospital Universitario Fundación Jiménez Diaz (IIS-FJD), Av. de los Reyes Católicos, 2, Madrid 28040, Spain; Biomedical Network Research Centre On Rare Diseases (CIBERER), Instituto de Salud Carlos III, Monforte de Lemos, 3-5, Madrid 28029, Spain; Departamento de Genética y Genómica, Hospital Universitario Fundación Jiménez Diaz (IIS-FJD), Av. de los Reyes Católicos, 2, Madrid 28040, Spain; Biomedical Network Research Centre On Rare Diseases (CIBERER), Instituto de Salud Carlos III, Monforte de Lemos, 3-5, Madrid 28029, Spain; Hereditary Endocrine Cancer Group, Human Cancer Genetics Program, Spanish National Cancer Research Center (CNIO), Melchor Fernández Almagro, 3, Madrid 28029, Spain; Biomedical Network Research Centre On Rare Diseases (CIBERER), Instituto de Salud Carlos III, Monforte de Lemos, 3-5, Madrid 28029, Spain; Hereditary Endocrine Cancer Group, Human Cancer Genetics Program, Spanish National Cancer Research Center (CNIO), Melchor Fernández Almagro, 3, Madrid 28029, Spain; Genetics Laboratory, Center for Genomic Medicine, Parc Taulí Hospital Universitari, Institut d’Investigació i Innovació Parc Taulí (I3PT-CERCA), Universitat Autònoma de Barcelona, Plaça Torre de l’Aigua, s/n, Sabadell 08208, Spain; Genetics Laboratory, Center for Genomic Medicine, Parc Taulí Hospital Universitari, Institut d’Investigació i Innovació Parc Taulí (I3PT-CERCA), Universitat Autònoma de Barcelona, Plaça Torre de l’Aigua, s/n, Sabadell 08208, Spain; Molecular Genetics Unit, Consorci Sanitari de Terrassa, Ctra. Torrebonica, S/N, Terrassa 08227, Spain; Molecular Genetics Unit, Consorci Sanitari de Terrassa, Ctra. Torrebonica, S/N, Terrassa 08227, Spain; Department of Clinical and Molecular Genetics, Vall d’Hebron Barcelona Hospital Campus, Vall d’Hebron Hospital Universitari, Pg. de la Vall d’Hebron, 119, Barcelona 08035, Spain; Medicine Genetics Group, Vall d’Hebron Institut de Recerca (VHIR), Vall d’Hebron Barcelona Hospital Campus, Vall d’Hebron Hospital Universitari, Pg. de la Vall d’Hebron, 119, Barcelona 08035, Spain; Centro de Investigación Biomédica en Red Cáncer (CIBERONC), Instituto de Salud Carlos III, Monforte de Lemos, 3-5, Madrid, 28029, Spain; Biomarkers and Personalized Approach to Cancer Group (BioPAC), Ramón y Cajal Health Research Institute (IRYCIS), Ctra. Colmenar Viejo, Km. 9,100, Madrid 28034, Spain; Fundación Centro Médico de Asturias, José María Richard Grandío, s/n, Oviedo, Asturias 33193, Spain; Cancer Genetic Counseling Unit, Medical Oncology Department, Elche General University Hospital, Almazara, 11, Elche 03203, Spain; Centro de Investigación Biomédica en Red Cáncer (CIBERONC), Instituto de Salud Carlos III, Monforte de Lemos, 3-5, Madrid, 28029, Spain; Program in Cancer Research, Institut Hospital del Mar d’Investigacions Mèdiques, Dr. Aiguader, 88, Barcelona 08003, Spain; Josep Carreras Leukemia Research Institute, Ctra de Can Ruti, Camí de les Escoles, s/n, Barcelona 08916, Spain; Hereditary Cancer Program, Catalan Institute of Oncology, Institut d’Investigació Biomèdica de Bellvitge-IDIBELL-ONCOBELL, L’Hospitalet de Llobregat, Barcelona 08908, Spain; Centro de Investigación Biomédica en Red Cáncer (CIBERONC), Instituto de Salud Carlos III, Monforte de Lemos, 3-5, Madrid, 28029, Spain; Hereditary Cancer Program, Catalan Institute of Oncology, Institut d’Investigació Biomèdica de Bellvitge-IDIBELL-ONCOBELL, L’Hospitalet de Llobregat, Barcelona 08908, Spain; Centro de Investigación Biomédica en Red Cáncer (CIBERONC), Instituto de Salud Carlos III, Monforte de Lemos, 3-5, Madrid, 28029, Spain

## Abstract

Accurate classification of genetic variants is crucial for clinical decision-making in hereditary cancer. In Spain, genetic diagnostic laboratories have traditionally approached this task independently due to the lack of a dedicated resource. Here we present SpadaHC, a web-based database for sharing variants in hereditary cancer genes in the Spanish population. SpadaHC is implemented using a three-tier architecture consisting of a relational database, a web tool and a bioinformatics pipeline. Contributing laboratories can share variant classifications and variants from individuals in Variant Calling Format (VCF) format. The platform supports open and restricted access, flexible dataset submissions, automatic pseudo-anonymization, VCF quality control, variant normalization and liftover between genome builds. Users can flexibly explore and search data, receive automatic discrepancy notifications and access SpadaHC population frequencies based on many criteria. In February 2024, SpadaHC included 18 laboratory members, storing 1.17 million variants from 4306 patients and 16 343 laboratory classifications. In the first analysis of the shared data, we identified 84 genetic variants with clinically relevant discrepancies in their classifications and addressed them through a three-phase resolution strategy. This work highlights the importance of data sharing to promote consistency in variant classifications among laboratories, so patients and family members can benefit from more accurate clinical management.

**Database URL**: https://spadahc.ciberisciii.es/

## Graphical Abstract

**Figure:**
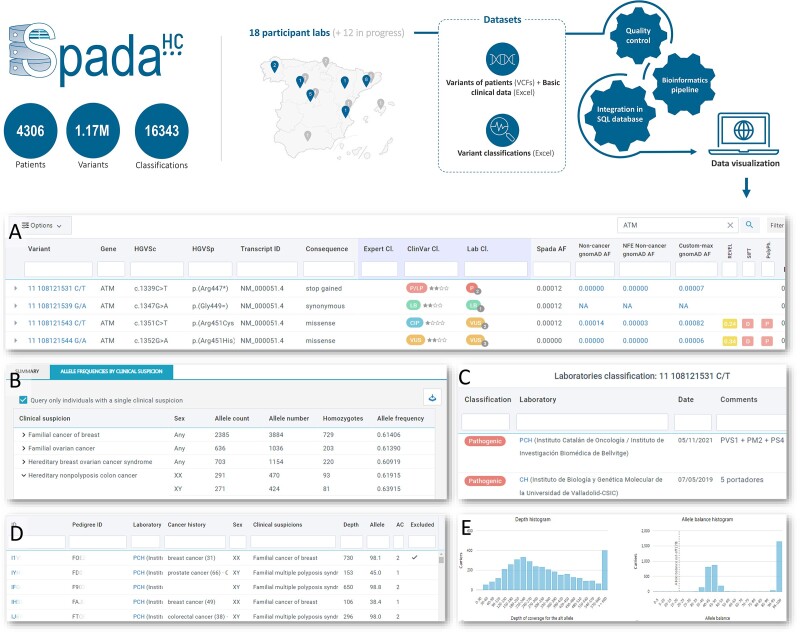
Overview of SpadaHC and its main views. (A) List of existing variants in SpadaHC (in the image, search for the ATM gene). The ‘Expert Cl.’ column shows the classification made by a group of experts; the ‘Lab Cl.’ column shows a summary of the classifications made by the laboratories. (B) Allele frequency of a variant in the SpadaHC population according to clinical suspicion and sex. (C) Classifications provided by the laboratories for a variant. (D) List of patients carrying a variant. (E) Histogram showing the coverage and frequency (allele balance) with which the variant was detected in carrier patients.

## Introduction

The accurate classification of genetic variants is key to enable informed clinical decision-making and to advance the understanding of hereditary cancer. The guidelines laid down by the American College of Medical Genetics and Genomics (acMG) and the Association of Molecular Pathology (AMP) provided a structured framework to categorize variants as benign (B), likely benign (LB), of uncertain significance (VUS), likely pathogenic (LP) or pathogenic (P) (1). In particular, the detection of likely pathogenic or pathogenic variants in clinically actionable genes is essential for the clinical management of the patients and their relatives. It allows for the individualization of cancer risk assessment, the establishment of specific surveillance measures, the use of appropriate targeted treatments and reproductive counseling. However, classifying variants can be challenging, as it requires collecting data from multiple sources. This process typically involves obtaining population frequencies from public databases, reviewing functional and case-control studies reported in the scientific literature, running *in silico* predictors and analyzing familial phenotypes and co-segregation.

Some of the issues associated with classification of variants are the disparities in the application of classification guidelines, which are frequently updated, or the use of private variant evidence to apply these rules (2, 3). These issues can cause differences in variant classification between laboratories, leading to inequalities in patient counseling and management, since genetic testing outcomes directly impact clinical decisions for patients. Interestingly, a significant obstacle to improving the uniformity of variant classification is the isolation of variant data within individual laboratories. In this context, data sharing is essential for achieving concordance of variant classification among laboratories (4). Professional organizations, such as the ACMG, have included the concept of variant sharing in their best-practice guidelines (5). Sharing data from multiple patients improves the evidence for genetic disease causality, increasing the statistical power of analyses and contributing to a more robust interpretation of variants supported by group consensus.

The need for collaborative initiatives on variant classification has also been underlined by projects from several countries such as CanVIG-UK in UK (6), MOLGENIS in the Netherlands (7), COGR in Canada (8, 9), Shariant in Australia (10) or MGeND in Japan (11). These initiatives highlighted the power of pooling data from diverse sources to enhance the accuracy and consistency of genetic variant classification. For example, the Canadian Open Genetics Repository (COGR) resolved 51.9% of variant classification discrepancies across 12 laboratories according to their two-tier model, while the Shariant platform did so for 42.9% of their medically significant discrepancies between 11 Australian laboratories (3, 10).

In Spain, laboratories have rarely shared variant data and classifications due to the lack of a dedicated resource. To promote data sharing, collaboration, improvement and concordance of variant classifications, we launched SpadaHC (SPAnish variant DAtabase for Hereditary Cancer), a national platform that enables Spanish genetic diagnostic laboratories to share variant classifications and patient variants in hereditary cancer genes. SpadaHC is the result of a nationwide effort promoted by the Biomedical Research Networking Center (CIBER) through the Oncology area (CIBERONC), with the participation of the Spanish Society of Human Genetics (AEGH), the Spanish Society of Medical Oncology (SEOM) and 18 genetic diagnostic laboratories. The data shared in SpadaHC allowed us to identify clinically relevant discrepancies between Spanish laboratories, and we implemented a three-phase methodology to address these discrepancies.

## Results

SpadaHC is a database for sharing genetic variants in hereditary cancer genes in the Spanish population. Spanish genetic diagnostic laboratories can submit two types of datasets (Graphical Abstract): (i) variant classifications through an Excel file, and (ii) variants of individuals [Variant Calling Format (VCF) files] along with basic clinical information of patients in Excel format. SpadaHC checks the submitted data, processes them through a bioinformatics pipeline, integrates them into the database and displays them on the SpadaHC website. This allows users to access the classifications provided by each laboratory and population frequencies based on multiple criteria.

### Available data

SpadaHC v1.62.0 (March 2024) included 57 datasets of classifications and 6 datasets of individual’s variants submitted by 18 Spanish laboratories. In total, SpadaHC stored 1.17 million variants in 225 genes from 4306 patients with hereditary cancer suspicion. The Spanish laboratories had shared 16 343 classifications through datasets of variant classifications. In sum, SpadaHC managed 21 397 unique variants, with 10 671 of the unique variants being classified by one or more laboratories.

### Features

#### Open and restricted access

SpadaHC contains open data, available to any user, and restricted data, available to registered users from Spanish laboratories or external researchers with granted access for specific projects. Unregistered users can only access de-identified genetic variant data, including population frequencies based on clinical suspicion of hereditary cancer, sex or laboratory. Registered users can also access personal data: the variants of an individual, cancer history, pseudo-anonymized identifiers and the classifications provided by the laboratories. This authorization approach enables SpadaHC to protect personal data while making the remaining information accessible to any user.

#### Flexible dataset submission format

Registered users with specific permissions can submit two types of datasets: variant classifications and variants of individuals. SpadaHC assesses that submitted Excel files fulfill the exact expected format. However, SpadaHC implements extensive flexibility to deal with these formats. Instead of requiring a single specific Excel format for all laboratories, SpadaHC requires a custom format for each laboratory (Supplementary File S1). This allows for adaptation to the format that is already being generated in the laboratory’s diagnostic routine. Consequently, the order of columns, column names and expected values may vary across laboratories, but SpadaHC will process and normalize them later.

#### Automatic identifier pseudo-anonymization

To maintain patient confidentiality, SpadaHC automatically pseudo-anonymizes individual and family identifiers provided by laboratories. Only registered users can access the pseudo-anonymized identifiers. The correspondence between the original identifier and the pseudo-anonymized one is stored in an internal database table that is accessible only to the administrators. In situations where re-identification of the patient is necessary due to a real and concrete danger to the safety or health of a person or group of people, or to ensure adequate healthcare, the ethics committee of the patient’s center must approve the re-identification of the patient.

#### VCF quality control

SpadaHC pipeline implements hard filters for each submitted VCF. Specifically, it excludes variants with FILTER field different from PASS, genotype equal to 0/0, allele balance lower than 0.2 or depth coverage lower than the custom threshold defined by the submitter (minimum is 10). Variants outside the gene panel regions file are also filtered out.

SpadaHC conducts various tests afterwards to verify the quality of the VCF data entered into the database. Specifically, SpadaHC detects whether a sample has far more variants than expected (noisy sample), far less variants than expected (empty sample), shares a high number of variants with another variant (duplicated sample) or *in silico* predictor estimates a kinship relationship with another sample in SpadaHC (see Methods). Noisy, empty or duplicated samples are not inserted into the database. Samples with a kinship relationship are entered into the database, but only one sample from each group of related samples is included in the allele frequency calculation.

#### Variant normalization

Accurate normalization of variant names is essential for processing and integrating coherent data into the database. Deletions and insertions can be annotated at multiple locations when they appear within repeated regions. SpadaHC normalizes all submitted variants as follows. Genomic positions are left-normalized, meaning that the most 5ʹ representation is used when referring to DNA. However, the coding DNA HGVS nomenclature follows 3ʹ normalization (12), a format commonly used by the clinical community. Therefore, SpadaHC uses right-normalized coding DNA HGVS nomenclature, and *in silico* predictors are computed after this normalization.

#### Liftover between genome builds

SpadaHC supports exploring variants in both GRCh37 and GRCh38 genome assemblies. To date, all submitted variants in SpadaHC were called using the GRCh37 assembly. SpadaHC obtains the GRCh38 coordinates by, first, calling CrossMap to lift over genome coordinates and, second, checking that the genomic reference base remains the same at the lifted over position. If any of the two steps fails, the variant will not have representation on GRCh38 assembly.

#### Search and explore data

SpadaHC allows exploring data with flexibility. The initial search option (Supplementary Figure S1) enables querying variants by gene symbol, coding DNA HGVS name, variant genomic definition and chromosomal region in the GRCh37 or GRCh38 genome assemblies. Moreover, queried variants can be restricted to a certain group of individuals by any combination of criteria including sex, clinical suspicion, submitter laboratory, gene panel, sequencing platform, aligner and variant caller used to generate the corresponding VCF file (Supplementary Figure S2).

The search action results in an adjustable, rich and user-friendly table of variants with up to 38 annotation columns (Graphical Abstract A). Specifically, users can access variant genomic definition, RefSeq transcript version, coding DNA and protein HGVS names, consequence predicted by VEP, expert group and laboratory classifications, weekly updated ClinVar classification, exon or intron number, amino acid change, SpadaHC allele frequency including allele count and allele number, gnomAD allele frequencies, REVEL/SIFT/PolyPhen-2 pathogenicity prediction, SpliceAI and MaxEntScan splicing prediction and co-located known variants. The table can be sorted and filtered by any number of columns, unfolded to access annotation in other transcripts (Supplementary Figure S3) and downloaded into an Excel file.

#### Variant classifications

SpadaHC provides access to variant classifications from three sources: submitting laboratories, the ClinVar database and expert groups. By clicking on any laboratory classification, SpadaHC displays details such as classification date, reasoning and, interestingly, the submitter’s name and email contact so that questions about the classification can be addressed (Graphical Abstract C). The ClinVar Classification field shows the weekly updated classification from ClinVar along with the level of assessment. Clicking on the ClinVar classification opens a new tab to the variant classification page in ClinVar. Finally, the expert group classification shows the classification given by a particular group of experts, namely Spanish researchers and clinicians engaged in the study of a gene or clinical suspicion, as exemplified by the work published by Feliubadaló *et al*. (13). Clicking on the expert group classification will show the details submitted by the expert group (Supplementary Figure S4).

#### Automatic discrepancy notification

Registered users can receive notifications via email when new classifications are added or updated on variants of their interest (Supplementary Figure S5). Users can flexibly define which are their variants of interest, that is, any variant that their laboratory classified in a certain way or is located within a user-defined list of genes. Users can also define the events that trigger the notifications, specifically, when variants of interest are newly classified into a user-defined group by any other laboratory or by ClinVar. These preferences are stored in the SpadaHC database. Consequently, upon the submission of a new laboratory classification to SpadaHC or the updating of a ClinVar classification, SpadaHC automatically checks the user preferences and sends a customized email, which lists the variants of interest.

#### Variant frequencies

The SpadaHC variant details view displays allele frequencies aggregated by clinical suspicion, sex and submitter laboratory (Graphical Abstract B). Furthermore, when querying variants using the main search option, SpadaHC will also present frequencies based on any VCF-related metadata field, such as clinical suspicion, sex, submitter laboratory, gene panel, sequencing platform, aligner and variant caller.

#### Variant details and individual view

The variant details view shows all the data available in SpadaHC for a specific variant (Supplementary Figure S6). In addition to the allele frequency tables mentioned above, the view provides a list of variant carriers (Graphical Abstract E) along with histograms of depth and allele balance (Graphical Abstract D). Registered users can click on any of the carriers and a new patient view will open with all the individual’s variants. The variant details view also provides other data such as laboratory, expert group and ClinVar classifications, context of the genome reference sequence, PubMed annotation (14), links to the variant in the Beacon Network (https://beacon-network.org) and VarSome (15), and the links to explore the genomic region in Ensembl (16) and UCSC browser (17). Both the variant details and individual views can be easily shared with other users via their URL.

### Use of SpadaHC to resolve classification discrepancies

**Figure 1.**
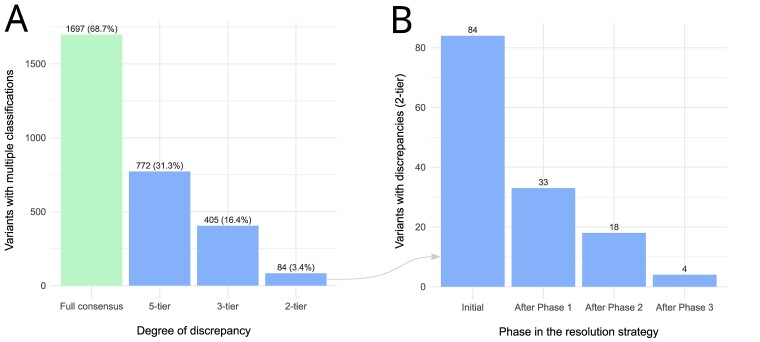
(A) Variants with multiple classifications in SpadaHC v1.21.0 and degree of discrepancy according to the five-tier, three-tier and two-tier models. (B) Number of variants with clinically relevant discrepancies according to the two-tier model after each phase of the resolution strategy.

In version 1.21.0 of SpadaHC (July 2023), laboratories had submitted classifications for 10 035 unique variants. Of these, 2469 were classified by at least two laboratories. Out of the 2469 variants with multiple classifications, 1697 (68.7%) had full consensus (Figure 1 A). While 31.3% and 16.4% showed discrepancies according to the five-tier and three-tier models (9), respectively, only 84 variants (3.4%) showed clinically significant discrepancies according to the two-tier model. To address the latter clinically relevant discrepancies, we implemented a three-phase strategy (see Methods, Figure 1 B). In the first phase, laboratories made a total of 93 variant revisions, with 51 out of 84 (60.7%) variant discrepancies being resolved. The remaining 33 variants with discrepancies were addressed in Phase 2, during which 15 (45.4%) were resolved after 52 revisions by the laboratories. In the third phase, laboratories resolved 14 variants with discrepancies (77.8%) through email and remote meeting discussions. Only four variants remained discordant after concluding the discrepancy resolution process (Supplementary Table S1): NM_003002.4:c.148C > G in *SDHD*, NM_032043.3:c.2990_2993del in *BRIP1*, NM_000551.4:c.376 G > A in *VHL* and NM_058216.3:c.965 + 5 G > A in *RAD51C*.

## Discussion

Variant classification is a complex task that requires collecting data from various sources. Since no dedicated resource existed in Spain, genetic diagnostic laboratories typically approached this task independently. Here we present SpadaHC, a dedicated resource for sharing genetic variants in hereditary cancer genes and their interpretation among Spanish genetic diagnostic laboratories.

Since its release in 18 May 2023, laboratory members have integrated SpadaHC, with another 12 in the process of joining. The participation of numerous Spanish laboratories has resulted in a significant contribution of data, including 1.17 million variants from 4306 patients and 16 343 variant classifications. This data sharing effort is valuable for improving knowledge of these variants in the Spanish population.

SpadaHC implements features for collecting, analyzing, annotating and exploring genetic variants. The platform offers open and restricted access, where only registered users can access personal and laboratory classification data, while the rest is public to any user. Furthermore, the flexible dataset submission system allows SpadaHC to adapt to the specific format of each laboratory while maintaining a strict parsing process. It is important to note that in the event a laboratory necessitates a new customized format, no changes to the code are required. This is because the format is defined in a JavaScript Object Notation field in the database. In addition, the user interface enables users to explore genetic variants in a variety of ways, benefiting from the ability to filter and sort by any number of criteria in the 38-column main table. Additionally, by receiving automatic notifications when a variant of interest is classified, users have an efficient tool to quickly track clinically relevant changes that may result in modifications to the patient’s clinical management. Furthermore, variant frequencies calculated from patients included in SpadaHC provide a useful resource for understanding how frequencies differ between sexes and/or clinical suspicions. Having the allele count for each variant can aid in identifying variants of interest, as enrichment in cases is a crucial factor in indicating variant pathogenicity.

The laboratory, ClinVar and expert group classifications are of particular interest to users. These classifications are summarized in just a few pixels and can be filtered or sorted as needed (Graphical Abstract A). Additional details can be obtained by clicking on any classification. The detailed view of laboratory classifications includes access to the email of the variant classification submitter, which facilitates cooperation between laboratories. Additionally, the weekly update of ClinVar classifications was a requirement in the design of SpadaHC in order to make it useful; otherwise, users may ignore this field when it became outdated.

SpadaHC was developed considering the FAIR (Findable, Accessible, Interoperable, Reusable) principles (18). All datasets and individuals submitted to SpadaHC are assigned with a unique persistent identifier, and datasets of variants of individuals contain rich metadata. These features result in better findability. For its part, the use of the HTTPS protocol along with the authorization system benefits accessibility while protecting the personal data from unauthorized users. Regarding interoperability, SpadaHC uses standardized notations such as HGVS variant nomenclature and RefSeq transcript references. Additionally, SpadaHC supports the standard VCF as input, and employs specific controlled vocabulary, the MedGen ontology, when referring to clinical suspicions. Finally, the rich metadata, including provenance information, along with the documentation available on the SpadaHC site, contributes to better reusability.

Some limitations in SpadaHC should be noted. The platform needs the laboratories to actively submit the variants and classifications. Instead, the ideal approach would be having the same laboratory management information system (LIMS) (19) in all laboratories and implementing a feature to automatically submit the found variants and classifications to SpadaHC. This way, data would be more quickly available in SpadaHC while limiting human errors on dataset submissions. Of course, this approach would require significant time and monetary resources, especially considering the challenge of involving many hospitals under different management and switching to a new LIMS in a demanding diagnostics routine context. Another limitation in SpadaHC is the heterogeneity of the submitted VCFs between laboratories. Each laboratory has its own diagnostics protocol, including aspects such as sequencing platform, gene panel or variant caller. Ideally, laboratories should submit the original sequencing files obtained from equal sequencing platforms and gene panels. This would allow SpadaHC to apply the same bioinformatics pipeline to all samples from the original files. Again, this approach would require significant resources and profound adaptation in the way genetic diagnostic laboratories operate. However, the aim of SpadaHC was not to force laboratories to undertake major changes, but to exploit existing laboratory data by adapting SpadaHC to their formats and characteristics. In any case, since SpadaHC requires rich metadata in the submission of datasets of variants of individuals (see Methods), users can flexibly restrict the data to explore a particular subset of patients according to different criteria. For instance, users can query variants called using a particular gene panel, aligner and variant caller.

### Use of SpadaHC to resolve classification discrepancies

Sharing data in SpadaHC allowed us to identify variants with discrepancies in their classifications. In particular, we identified clinically significant differences in 84 variants according to the two-tier model (9). Discrepancies across laboratories can be explained for multiple reasons. Spanish laboratories apply the classification guidelines independently, not always using gene-specific ones, and frequently work with evidence from their own patients. Also, many laboratories do not share most of their data with other laboratories or international databases. Additionally, evidence and classification guidelines may vary over time, favoring discrepancy in classifications made far in time, as previously suggested (20).

The aim of our three-phase strategy was to resolve the 84 clinically significant discrepancies in a cost-effective manner while minimizing the workload of the laboratories. To achieve this, we employed criteria from previous discrepancy resolution initiatives, including COGR (9), Shariant (10), CanVIG-UK (6) and the All of Us Research Program (AoURP) (21). Our approach followed more specially the cost-effective methodology presented by the AoURP, which prioritized reassessment by the laboratory with the oldest classification or the laboratory with an outlier classification. However, our strategy differs from the latter in several ways, including the order in which we applied the criteria, the scope of the outlier and oldest criteria and the use of email groups to discuss the most complicated variants in our third and final phase. Overall, the participating laboratories carried out 145 revisions using our discrepancy resolution strategy. This contrasts with the 285 revisions that would have been required if a brute-force strategy, where laboratories would have re-analyzed all discrepant variants, had been used.

Eighty out of the initial 84 (95.2%) discrepancies were resolved after Phase 3. This percentage is higher when compared to similar previous studies such as COGR, which resolved 51.9% and 81.2% of discrepancies according to the two-tier model in different works (3, 22), or Shariant, which resolved 42.9% of medically significant discrepancies (23). The percentage is also slightly lower than the work performed by the AoURP which resolved all discrepancies (21). In any case, the rate of discrepancy resolution may depend on several aspects such as the number of discrepant variants and the methodology used to address them. Also, these rates may depend on the genes in which variants are located. Some genes accumulate more knowledge on their associated risks, have clinically calibrated functional assays, a very specific or penetrant phenotype or possess gene-specific guidelines. Classifying variants on those genes is less prone to subjectivity and discrepancies can be addressed more easily. In our work, classification of four variants remained discordant, mostly due to different weight assigned to the same evidence.

### Future directions

#### Beacon network

The Beacon Network is an international collaborative effort aimed at creating a standardized infrastructure for sharing genomic variant data across disparate databases. In its first version, this initiative allows individual databases to implement a ‘beacon’ that responds with a simple ‘yes’ or ‘no’ when queried about the presence of a specific genetic variant within their dataset, without disclosing any sensitive information. We plan to join the Beacon Network in the future, so any researcher using this will be able to know if a particular variant is stored in SpadaHC, which will also result in better findability according to the FAIR principles.

#### Automated ClinVar submission

ClinVar is a reference repository for data on the interpretation of variants observed in clinical testing. We plan to implement in SpadaHC automated ClinVar submissions through the ClinVar submission API upon submitter laboratory approval. This automation may accelerate and increase the number of submissions to ClinVar from Spanish laboratories, while reducing the likelihood of submission errors or omissions.

## Methods

### Implementation

SpadaHC is a web-based database that follows a three-tier architecture consisting of a web server, a database server and a computation server. The web server is implemented using the model-template-views (MTV) architectural pattern supported by Django v3.2.5. Authorizations and invitations are managed using Django invitations v1.9.3 and Django allauth v0.45. The database server stores a PostgreSQL v14.4 relational database. The computation server runs a bioinformatics pipeline to verify, standardize and annotate genetic variants shared by laboratories. The pipeline is implemented using R v4.1.2, Variant Effect Predictor (VEP) v104 for GRCh37.p13 and GRCh38.p13 assemblies (24), Bioconductor v3.14 (25), bedtools v2.26.0 (26), bcftools v1.14 (27), vcftools v0.1.16 (28), SpliceAI v1.3.1 (29), REVEL v1.3 (30), CrossMap v0.5.4 (31), MaxEntScan (32, 33), gnomAD v2.1.1 (34) and weekly updated ClinVar classifications (35). SpadaHC can be accessed at https://spadahc.ciberisciii.es/ and the web code is publicly available in the Figshare repository (https://doi.org/10.6084/m9.figshare.25311124.v1).

The data privacy of patients is assured by a number of mechanisms. Only registered users have access to sensitive data, which is controlled by requiring a username and password. Furthermore, the web server has a demilitarized zone (DMZ) configuration and multiple Apache security modules. Firewalls have also been established at different levels through the architecture. Additionally, a number of security measures have been implemented in the web code, including cross-site scripting (XSS) protection, cross-site request forgery (CSRF) protection, SQL injection protection, clickjacking protection and the limitation of the size of files that can be uploaded to limit denial of service (DOS) attacks. Finally, the automatic identifier pseudo-anonymization, as explained in Features, ensures that patients cannot be identified through their identifiers.

### Laboratory and user registration

In order to participate in SpadaHC, Spanish laboratories are required to sign a data transfer agreement (DTA) contract together with CIBER. Additionally, those laboratories aiming to submit datasets with variants of individuals (VCFs) are asked to obtain a favorable evaluation from their ethics committee and required to get a signed informed consent of patients. Once the DTA is signed, the principal investigator of the group sends a document requesting the registration of a list of laboratory members. The SpadaHC management team reviews the request and sends an invitation to each requested user. This is done via email, which contains a unique link. The link is only valid for one use and has an expiry date.

Researchers and groups based abroad can also register by sending a request with a short project proposal through the SpadaHC website. Subsequently, the SpadaHC advisory board and steering committee review the request and, if approved, the SpadaHC management team sends an invitation to the users who requested to join.

### Dataset submission

Registered users with the appropriate permissions can submit two types of datasets to SpadaHC: variant classifications and variants of individuals. To submit a dataset of variant classifications, users must upload an Excel file containing the definition of the variant, including the genomic or cDNA position and transcript reference, classification, date of classification and reasoning. For variants that have already been classified by the submitted laboratory in SpadaHC, the most recent classification is shown based on the date of classification. To submit a dataset of variants of individuals, users must upload VCF files containing the variants, one per individual, along with basic clinical information of the patients in Excel format. The VCFs of a dataset must be homogeneous, meaning they must be generated using the same sequencing platform, read type, panel version, genome version, aligner and variant caller. This information is also submitted to SpadaHC to provide the dataset with rich metadata. The Excel file must include an individual identifier, sex and at least a clinical suspicion of hereditary cancer. Optionally, it may also include family identifier, cancer history, birth date and deceased status. SpadaHC supports several variant callers including VarScan2, Strelka2, GATK HaplotypeCaller, GATK UnifiedGenotyper, Torrent Variant Caller, VarDict and DNAscope. When referring to clinical suspicion of hereditary cancer, laboratories must use terms from the MedGen ontology (36), whose genetic disease terms are explicitly permitted by ClinVar. All datasets and individuals submitted to SpadaHC are labeled with a unique persistent identifier. Each dataset submission, either variant classifications or variants of individuals, results in a new incremental version of the database.

SpadaHC pseudo-anonymizes individual and family identifiers during the submission process. The new code is randomly generated using 36 uppercase alphanumeric characters. The code is six and five characters long for individuals and families, respectively.

### Allele frequency calculation

The allele frequency shown in SpadaHC (AF) is the result of dividing the allele count (AC) by the allele number (AN). The AN for a specific variant is calculated considering only the individuals having the corresponding genomic position covered, as indicated in the regions of interest file provided by the submitting laboratory. Also, when two or more individuals are related, either because they share the same family identifier or because the *in silico* prediction estimated a kinship relationship, only one individual is considered for AF calculation.

### Noisy, empty, duplicate and kinship tests

SpadaHC performs tests to detect whether a sample from a dataset of variants of individuals is noisy, empty, duplicated or has a kinship relationship with another sample in SpadaHC. To detect noisy samples, which are those with an unexpected high number of variants, we apply Tukey’s fences upper threshold (*k* = 5) to the distribution of the number of variants in each sample. Similarly, to detect empty samples, which are those with an unexpected low number of variants, we apply Tukey’s fences lower threshold (*k* = 4) to the same distribution. In addition, two samples are considered duplicates if the mean of p1 and p2 is greater than 0.9, where p1 is the percentage of variants in the first sample that were also called in the second sample, and p2 is the percentage of variants in the second sample that were also called in the first sample. Also, kinship relationships are identified (cutoff at 0.25) using the relatedness statistic described by Manichaikul *et al*. (37) and implemented in VCFtools (28). To ensure consistency, all tests are performed using the intersection of the compared regions, as VCFs may have been obtained from different targeted panels and may therefore span different regions.

### Main transcript selection

By default, SpadaHC displays annotations based on the main transcript of the gene, although more transcripts can be displayed. The selection of the main transcript is made as follows. When a variant is annotated on a single gene, the main transcript shown is the one that matches the first Locus Reference Genomic (LRG) transcript in that gene. If no LRG transcript is available for the gene, then the transcript shown is the one labeled as Canonical by the Ensembl VEP tool.

When a variant is annotated on multiple genes, the above criterion is used to select the candidate transcripts of each gene. The main transcript selected to be shown will be the one with the most severe consequence of the candidate transcripts.

### Use of SpadaHC to resolve classification discrepancies

Upon reaching SpadaHC version 1.21.0, we queried the database to identify variants with classification discrepancies based on the five-tier model (P, LP, VUS, LB, B), three-tier model (LP/P, VUS, LB/B) and two-tier model (LP/P being clinically actionable, VUS/LB/B being not) (9). We then focused on the 84 variants with classification discrepancies according to the two-tier model and implemented a three-phase strategy to resolve them. The strategy’s general criterion was to achieve a cost-effective balance by minimizing the number of laboratories assigned to review each variant. At the end of each of the three phases, all designated laboratories resubmitted their reviewed classifications, including reasoning, to SpadaHC for identification of any remaining discrepancies.

#### First phase

In the first phase, each variant was assigned to one or more laboratories according to the following algorithm:

1. If only two laboratories differed in their classification, the laboratory with the oldest classification reviewed its classification.
2. If more than two laboratories differed in their classification:
  - If a classification was an outlier, the laboratory that submitted that classification reviewed its classification. A classification was considered an outlier if one laboratory classified the variant in one group (actionable or non-actionable) and the remaining laboratories (at least two) classified it in the other group.
  - If two laboratories classified the variant in one group (actionable or non-actionable) and the remaining laboratories (at least two) classified the variant in the other group, one laboratory was randomly selected from the first group to review the classification.
3. For the remaining variants, all laboratories were invited to review their classifications.

#### Second phase

Phase 2 addressed variants with clinically relevant discrepancies that were not resolved in Phase 1. The algorithm used in this phase was as follows:

1. If a variant was assigned using point 1 (oldest classification) or 2 (outlier) in Phase 1, the remaining laboratories reviewed their classification.
2. For the variant addressed in Phase 1 via point 3, only one laboratory disagreed with the others, and this laboratory was assigned to carry out a further review.

#### Third phase

During Phase 3, we addressed variants that still had discrepancies after Phase 2.

1. For variants classified by up to three laboratories, we created an email group for each variant. In this group, each laboratory provided their evidence, and a discussion followed until a resolution was reached.
2. For variants classified by four or more laboratories, we held two video call meetings with all SpadaHC laboratories (the second one with a guest expert). There, the different laboratory members presented the evidence on which they based their classifications and, after a discussion, each laboratory made its decision.

## Supplementary Material

baae055_Supp

## Data Availability

SpadaHC is accessible at https://spadahc.ciberisciii.es/. The web code of SpadaHC can be found at the Figshare repository (https://doi.org/10.6084/m9.figshare.25311124.v1).
