# Supplementary material for: SpadaHC: a database to improve the classification of variants in hereditary cancer genes in the Spanish population
Source: Database (Oxford). 2024 Jul 4;2024:baae055. doi: 10.1093/database/baae055 (PMC11223915; doi:10.1093/database/baae055)
Supplement: baae055_Supp [file baae055_supp.zip › suppl_data/Supplementary Figures.pdf]

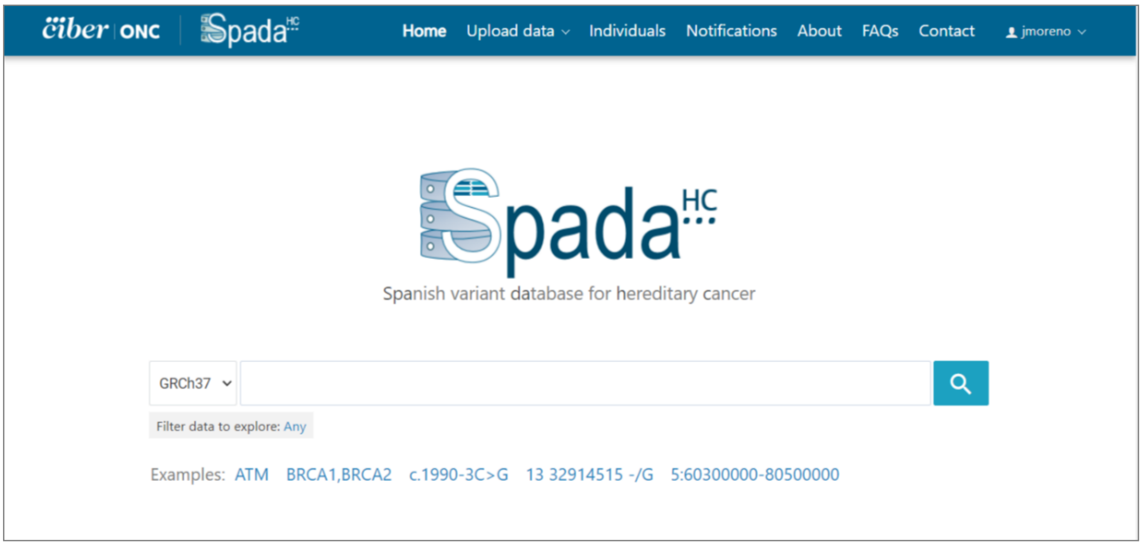

Supplementary Figure 1. Initial search option in SpadaHC at Home page.

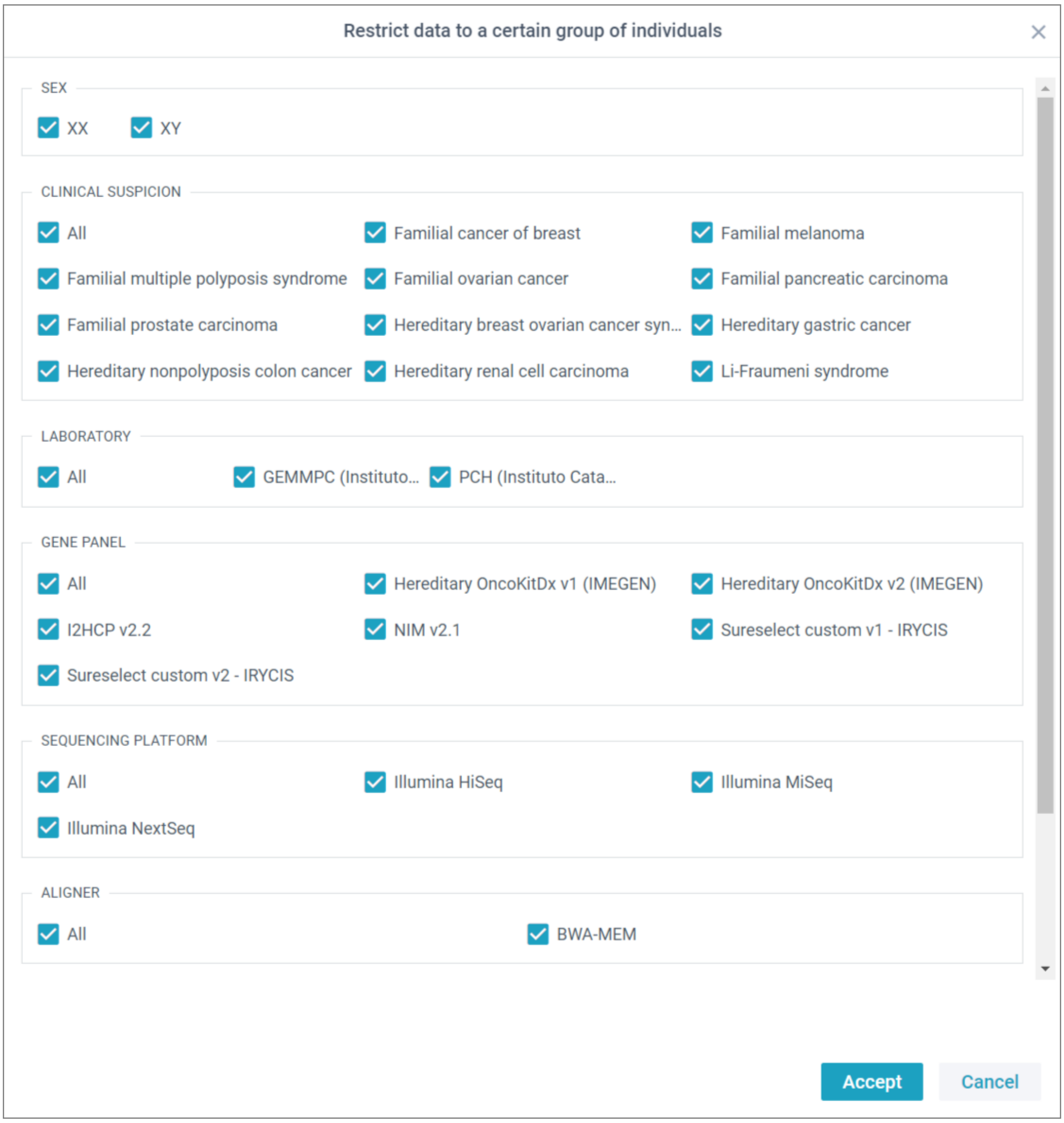

Supplementary Figure 2. Queried variants can be restricted to a specific group of individuals based on multiple criteria.

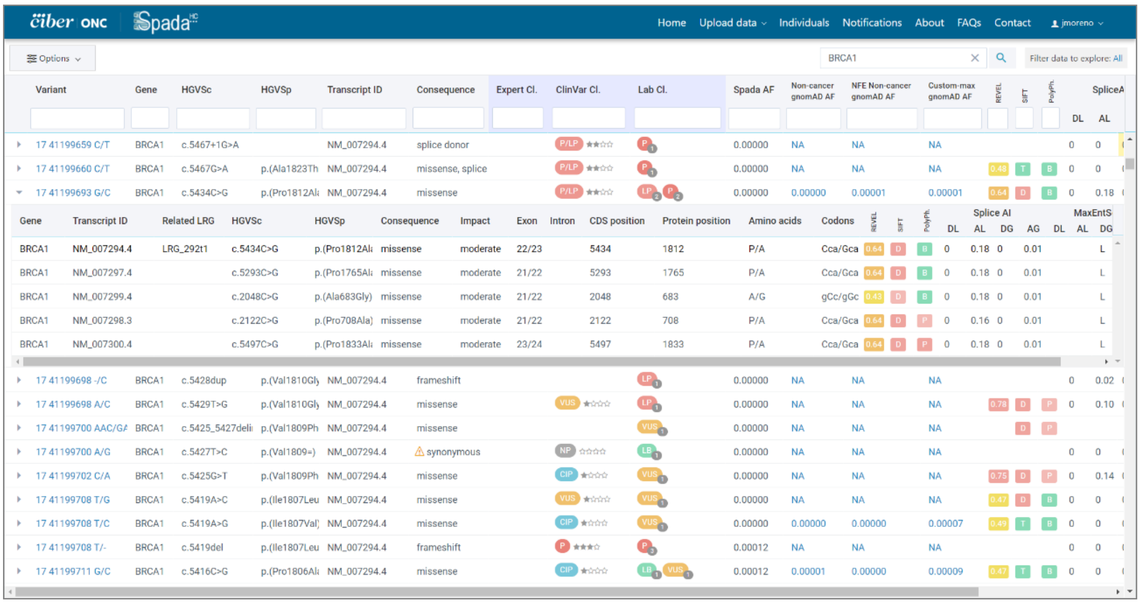

Supplementary Figure 3. Each row can be unfolded in the main results table to show annotation in other transcripts.

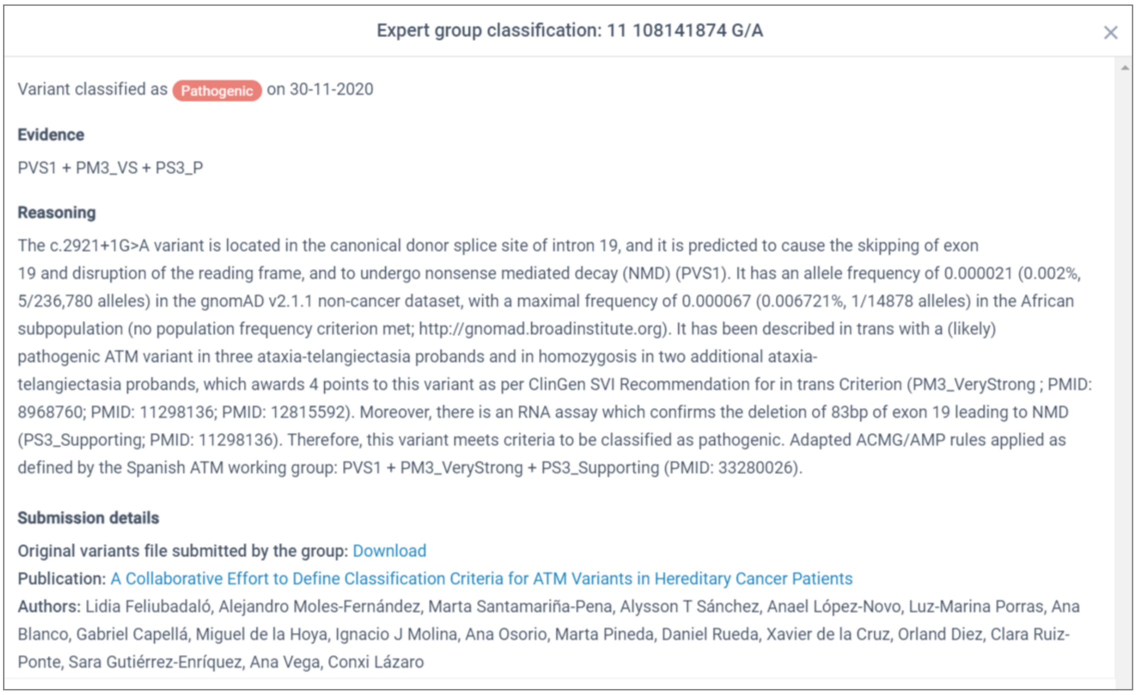

Supplementary Figure 4. Expert group classification details for a given variant, in this case variant 11 108141874 G/A.

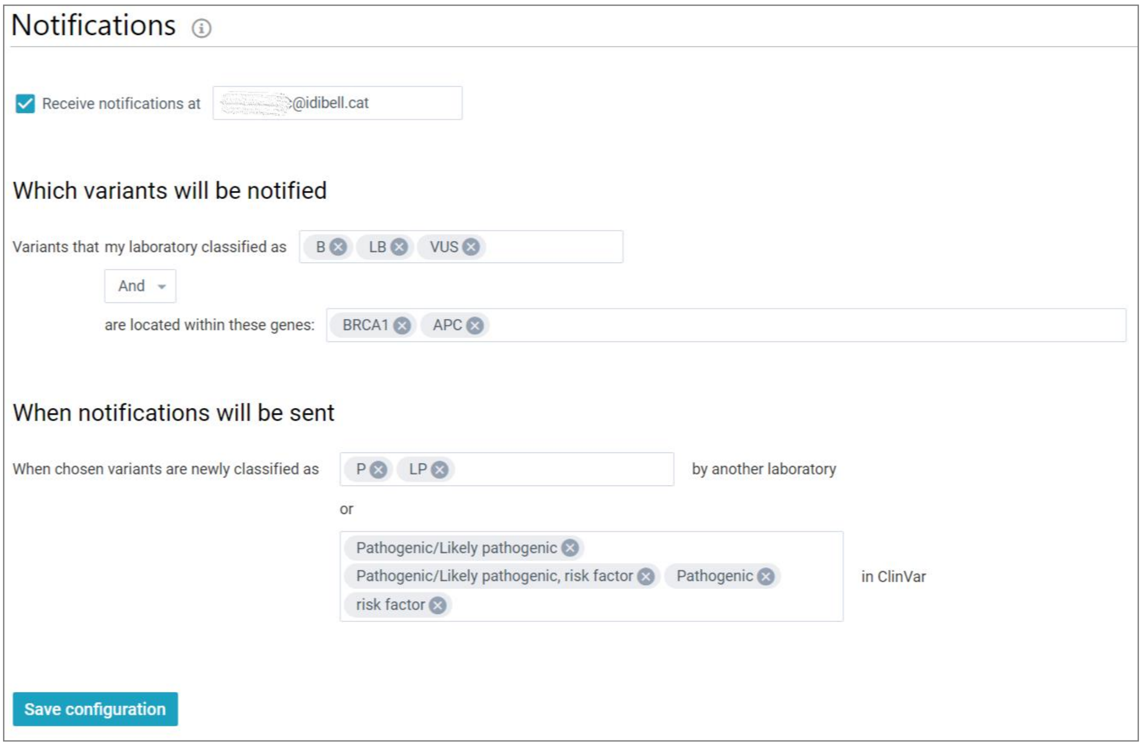

Supplementary Figure 5. Registered users can receive notifications via email when new classifications are added or updated on variants of their interest.

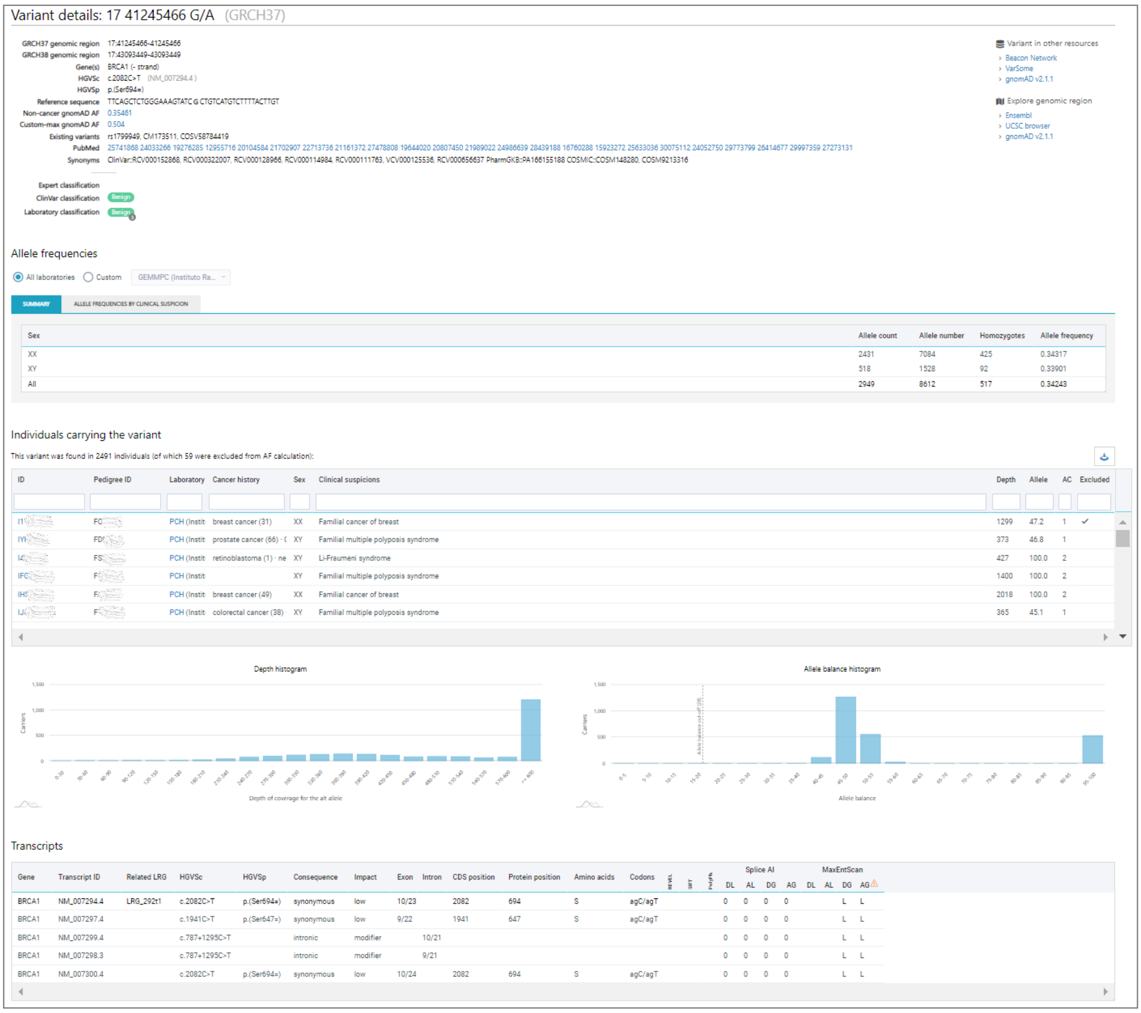

Supplementary Figure 6. Variant details view for variant 11 108151707 -/A.
