# Supplementary material for: SpadaHC: a database to improve the classification of variants in hereditary cancer genes in the Spanish population
Source: Database (Oxford). 2024 Jul 4;2024:baae055. doi: 10.1093/database/baae055 (PMC11223915; doi:10.1093/database/baae055)
Supplement: baae055_Supp [file baae055_supp.zip › suppl_data/Supplementary File 1.pdf]

Custom Excel format on dataset submissions

SpadaHC does not require a single specific Excel format for all laboratories. Instead, it adapts to the format that is already being generated in the laboratory's diagnostic routine. The information of how to adapt to each laboratory is defined in a JSON field in the database, so no changes of code are required to adapt to new formats.

Specifically, the JSON field defines:

- Mapping of expected column names.
- How these columns will be processed.
  - In case of dataset of classifications, user can submit variants either using NM:cDNA or genomic annotation.
  - In case of datasets of VCFs, JSON fields define how cancer history is stored (structured or free text field) and which values are expected in sex and exitus columns.
- Which values are expected. Some tables in the database define a set of expected values to be interpreted in a certain way. For example, a VUS classification can be submitted using any of these values: VUS / Variant of uncertain significance / Uncertain significance / Vus / vus / VSD / Vsd / vsd / UV / Uncertain Significance / VOUS /Clase 3 (VOUS)

Example of a JSON field for defining how to process Excel containing laboratory classifications

```json
{
	"COLUMNS": {
		"NM": "Transcript",
		"CDNA": "cDNA",
		"GENE": "Gene",
		"COMMENTS1": "Reasoning",
		"CLASSIFICATION": "Classification",
		"CLASSIFICATION_DATE": "Date"
	},
	"ASSEMBLY": "cDNA"
}
```

Example of a JSON field for defining how to process Excel containing clinical data from individuals for datasets of VCFs

```
{
	"OTHER": {
		"SEX_value_XX": "F",
		"SEX_value_XY": "M",
		"EXITUS_value_true": "T"
	},
	"COLUMNS": {
		"ID": "patient id",
		"SEX": "sex",
		"VCF": "VCF",
		"BIRTH": "birth date",
		"EXITUS": "exitus",
		"PEDIGREE": "Family ID"
	},
	"SUSPICION": {
		"names": ["clinical suspicion"]
	},
		"CANCER_HISTORY": {
		"TYPE": "text",
		"TEXT_COLUMN": "cancer history"
	}
}
```
