# Supplementary material for: SpadaHC: a database to improve the classification of variants in hereditary cancer genes in the Spanish population
Source: Database (Oxford). 2024 Jul 4;2024:baae055. doi: 10.1093/database/baae055 (PMC11223915; doi:10.1093/database/baae055)
Supplement: baae055_Supp [file baae055_supp.zip › suppl_data/Supplementary Table 1.docx]

**Supplementary Table 1. Discrepant variants after 3-phase resolution strategy**

| **Gene (transcript)** | **cDNA notation** | **Laboratory (class. date)** | **Class.** | **Reasonings** |
| --- | --- | --- | --- | --- |
| SDHD (NM_003002.4) | c.148C>G | Lab 1 (11/10/2023) | VUS | PS4_mod (identified in paraganglioma patients and families: Kung JT, Oh DK. In: The Endocrine Society's 93rd Annual Meeting & Expo. 2011 Jun 4-7; Boston. Abstract P2-664; Invitae internal data, as reported in ClinVar) + PM2_supp (1/251456 in gnomAD v2.1.1, non cancer dataset) + PP3 (SpliceAI no impact, p.(His50Asp) REVEL 0.75, PMID: 36413997). |
|  |  | Lab 2 (28/7/2023) | LPAT | Franklin. No other mutation in a patient with multiple H&N PPGLs. Likely pathogenic in ClinVar. 1/240000 alleles GnomAD |
| BRIP1 (NM_032043.3) | c.2990_2993del | Lab 1 (12/9/2023) | VUS | PVS1 (frameshift with a predicted alternate stop codon (p.(Thr997Argfs*61)). not expected to result in loss of function by NMD, but it removes >10% of protein. The downstream variant c.2992_2995del, causing a very similar frameshift, p.(Lys998Glufs*60), has been shown to impair the stability of the protein and the interaction with BRCA1. |
|  |  | Lab 2 (20/10/2023) | PAT | PVS1: frameshift LOF gene; PM2: not in gnomAD; PP5: PAT in ClinVar with 2 stars. Varsome PAT. Franklin LPAT. |
|  |  | Lab 3 (11/10/2023) | LPAT | Frameshift variant in the last exon of BRIP1 containing a BRCA1 interacting domain. It generates a new and aberrant sequence of 61 aa and a shorter protein, affecting about 20% of the protein. Other premature stop codons in the last exon of this gene are considered class 4 or 5. ClinVar: PAT 15 submitters, LPAT 6 submitters. Our case: CRC dx 38 with a MSH2 PAT mutation. |
| VHL (NM_000551.4) | c.376G>A | Lab 1 (17/10/2023) | VUS | SpliceAI no impact, p.(Asp126Asn) REVEL 0.716, (PP3, PMID: 36413997). 4/236956 in gnomAD v2.1.1, non cancer dataset. Variants in VHL are associated to 2 different phenotypes: autosomal dominant Von Hippel-Lindau disease and autosomal recessive familial erytrocytosis type 2/Chuvash polycythemia (caused by suspectedly hypomorphic missense or splicing variants that in heterozygosis sometimes cause some paragangliomas and pheochromocytomas, but not the whole VHL phenotype (PMID:24115288, PMID: 29891534). This variant has been has been identified in 5 individuals with a VHL-related phenotype (ICO internal data, Ambry Genetics, according to its report in ClinVar) (PP4_moderate). Reported in homozygosis in one individual with erythrocytosis and in heterozygosis together with different uncertain significance variants (c.548C>T p.S183L; c.162G>C, p.M54I) in two individuals affected with erythrocytosis and pulmonary arterial hypertension (PMID: 24729484, PMID:21454469, PMID:35734542, PM3). Functional studies of c.376G>A and c.548C>T showed intermediate levels of HIF‐1α and intermediate expression of its target genes (PMID: 21454469) (PS3_Supporting for an hypomorphic classification?). c.376G>A is classified as VUS, although with some evidence of possible hypomorphic function that would cause familial erytrocytosis type 2/Chuvash polycythemia. |
|  |  | Lab 2 (11/10/2023) | VUS | ClinVar: this variant is considered VUS for VHL and prob pat for recessively inherited erythrocytosis or polycythemia. (Ambry Genetics 2020, Gene Dx; Invitae). Other four submitters consider VUS for Von Hippel Lindau syndrome. Our 2 cases: 1) CRC and gastric cancer dx <50a; and 2) paraganglioma dx 44. |
|  |  | Lab 3 (28/7/2023) | PAT | LOVD, found in 2 patients with PPGL. Found in patients with PPGL and in patients with erythrocytosis (HH or compound Hh) |
| RAD51C (NM_058216.3) | c.965+5G>A | Lab 1 (11/10/2023) | LPAT | Intronic variant located close to a canonical splice site. Not in gnomAD v2.1.1, non cancer dataset (PM2_supporting). SpliceAI predicts the abolition of intron 7 splicing donor site. Three RNA studies, comprising patient RNA and minigene assays, showed the complete deletion of exon 7 (r.905_965del, p.Glu303Trpfs*41) (PMID: 33011440, 22725699, 33333735). This alteration disrupts the nuclear localization signal of RAD51C, necessary for proper cellular localization and resistance to mitomycin C (PMID: 12966089). The altered region also encompasses two positions where missense non-functional variants (p.(Thr336Pro) and p.(Asp348Val)) have been described (PMID: 37253112, PVS1 (RNA)). Identified in individuals with breast and/or ovarian cancer (internal data, PMID: 22725699). |
|  |  | Lab 2 (17/10/2023) | PAT | PVS1_Observed. (Experimental data shows skipping exon7 disrupting reading frame and predicted to undergo NMD). PS4_supp (present in affected cases and extremely rare in controls). PM2_supp (variant not found in non cancer gnomAD population) |
|  |  | Lab 3 13/10/2023) | LPAT | PM2_supp (1) + PVS1_O (RNA STUDIES, based on CanVar Guidelines) (8) |
|  |  | Lab 4 (11/10/2023) | LPAT | This alteration results in out-of-frame exon skipping and premature protein truncation (PMID: 22725699, PMID: 33333735 and others). |
|  |  | Lab 5 (2/10/2023) | VUS | PM2_P (absent in gnomAD v2.1.1 non-cancer), PVS1_S (Skipping exon 7, r.905_965del, p.Glu303Trpfs*41, minigene studies and lymphocyte culture with puromycin (PMID: 22725699, 33011440, 33333735). Co-ocurs in a patient with a PAT variant in PALB2. Reported in individuals with breast cancer and ovariant cancer (PMID: 22725699, 33011440). Gudelines: CanVIG-UK v2.7 + PMID: 36865205. |
